# Comparative study of ultrasound-guided paravertebral block versus intravenous tramadol for postoperative pain control in percutaneous nephrolithotomy

**DOI:** 10.1186/s12871-018-0479-7

**Published:** 2018-02-17

**Authors:** Zehra Hatipoglu, Ersel Gulec, Mediha Turktan, Volkan Izol, Atilla Arıdogan, Yasemin Gunes, Dilek Ozcengiz

**Affiliations:** 1 Faculty of Medicine, Department of Anaesthesiology and Reanimation, Cukurova University, 01250 Adana, Turkey; 2 Faculty of Medicine, Department of Urology, Cukurova University, Adana, Turkey

**Keywords:** Paravertebral block, Tramadol, Postoperative analgesia

## Abstract

**Background:**

Percutaneous nephrolithotomy (PCNL) is a minimally invasive surgical procedure for renal calculi, and nephrostomy tubes lead to postoperative pain after PCNL. Regional techniques (e.g., epidural analgesia and peripheral blocks) and opioids are applied for postoperative pain treatment. The aim of this study was to compare effectiveness of ultrasound-guided paravertebral block (PVB) and tramadol on postoperative pain in patients who underwent PCNL.

**Method:**

Fifty-three patients were included in this prospective randomized study. The patients were allocated into two groups: the PVB group (group P, *n* = 26) and the tramadol group (group T, *n* = 27). All patients were administered standard general anaesthesia. Ultrasound-guided PVB was performed at the T11- L1 levels using 0.5% bupivacaine for a total dose of 15 mL in group P. Patients in group T were intravenously administered a loading dose of 1 mg/kg tramadol. Patients in both groups were given patient-controlled analgesia. Haemodynamic parameters, visual analogue scale (VAS) scores, side effects, and complications, tramadol consumption and additional analgesic requirements of the patients were recorded after surgery.

**Results:**

Haemodynamic parameters were statistically similar between the groups. The VAS in group P were statistically lower than in group T. In the 24-h period after surgery, total PCA tramadol consumption was statistically lower in group P than in group T. The use of supplemental analgesic in group T was higher than in group P.

**Conclusions:**

Ultrasound-guided PVB was found to be an effective analgesia compared to tramadol, and no additional complications were encountered.

**Trial registration:**

ClinicalTrials.gov, NCT02412930, date of registration: March 27, 2015, retrospectively registered.

## Background

Percutaneous nephrolithotomy (PCNL) is a minimally invasive surgical procedure commonly used to treat renal calculi. A nephrostomy tube is usually inserted after PCNL to provide adequate urinary drainage, haemostasis, and access for possible complications. However, nephrostomy tubes causing postoperative pain and prolonged hospitalization can cause patient dissatisfaction [1].

Intravenous opioids, local anaesthetic infiltration, and peripheral nerve blocks are analgesia techniques used to control postoperative pain in adult patients undergoing PCNL [2–6]. Intercostal nerve blocks and paravertebral blocks (PVBs) have been performed as peripheral nerve blocks in several studies related to PCNL [4–6]. Paravertebral block provides effective analgesia with fewer systemic side effects (e.g., hypotension, nausea and vomiting) than an epidural block or opioids [7, 8]. Ultrasound-guided PVB allows real-time visualization of the needle, target nerve, adjacent anatomical structures, and applied local anaesthetic, thereby contributing to reducing not only the incidence of complications (e.g., pneumothorax and vascular puncture) and the failure rate but also local anaesthetic dosages [9].

Tramadol is a weak μ-opioid receptor agonist that inhibits noradrenaline and serotonin uptake, and it is widely used for postoperative pain management [10]. However, it may be inadequate for pain relief. In such a case, multimodal analgesia techniques or peripheral blocks may be preferential for analgesia management.

This study was designed to assess the analgesic efficacy of an ultrasound-guided PVB versus tramadol intravenous (IV) patient controlled analgesia (PCA) for PCNL. The primary endpoint was the postoperative pain level based on the visual analogue scale (VAS) scores of patients. The secondary endpoints were tramadol consumption and additional analgesia requirements of the patients in the postoperative period.

## Methods

This prospective randomized study was approved by the Local Ethics Committee (no: 5/8). In addition, the study protocol is registered at ClinicalTrials.gov (NCT02412930). Written informed consent was obtained from all patients.

Fifty-three patients scheduled for PCNL were included in the study. The inclusion criteria of this study were an age of 18-70 years and an American Society of Anaesthesiologists classification of I or II. The exclusion criteria were coagulation abnormalities, spinal deformities, cutaneous infection at the injection site, a known allergy to drugs, and patient refusal. The patients were randomly allocated into two groups: the PVB treated group (group P, *n* = 26) and the tramadol treated group (group T, *n* = 27). Randomization was performed based on a computer-generated randomized list before surgery.

The patients were given no premedication. All patients received standard monitoring, including electrocardiography, non-invasive arterial blood pressure, and peripheral oxygen saturation (SpO_2_) (Draeger-Primus Anaesthesia Device Monitor, Draeger Medical Systems, Inc., Denver, MA, USA) in the operating room. After the intravenous induction of anaesthesia with propofol (2 mg/kg), rocuronium bromide (0.5 mg/kg), and fentanyl (2 μg/kg), the patients were intubated with a suitable endotracheal tube. Anaesthesia maintenance was provided with 1-2% sevoflurane and a gas mixture of 60% nitrous oxide and 40% oxygen, and controlled ventilation was applied (Draeger-Primus Anaesthesia Device Monitor, Draeger Medical Systems, Inc., Denver, MA). Fluid resuscitation was performed with 0.9% NaCl (5-10 mL/kg). After the insertion of a urinary catheter, the patients were placed in the prone position.

Before the start of surgery, a PVB was performed under ultrasound guidance (MyLab™ Five, Esaote, Genoa, Italy) at the T_11_, T_12_ and L_1_ levels using 0.5% bupivacaine at a total dose of 15 mL in group P. After the injection site was cleaned with a 10% povidone-iodine solution, it was covered with sterile drapes. At the same time, a linear assay probe (7.5-12 MHz) was prepared under sterile conditions. The paravertebral space in the ultrasound view was defined as the area between the costotransverse ligament, pleura, and transverse process. A 22-gauge insulated echogenic needle (Stimuplex; B. Braun Medical Inc., Bethlehem, PA, USA) was advanced in the vertical-to-caudal direction using the in-plane technique. After the needle entered the paravertebral space, 5 mL of 0.5% bupivacaine was injected in each dermatome level, controlled by aspiration. The spread of the local anaesthetic was confirmed by anterior movement of the pleura in the paravertebral space. All blocks were performed by the same experienced anaesthesiologist.

Patients in group T were intravenously administered a loading dose of tramadol at 1 mg/kg 45 min before the end of the surgery. Patients in both groups received PCA. The PCA (CADD Legacy PCA pump, Smiths Medical MD, Inc., St. Paul, MN) was prepared with 400 mg tramadol in 100 mL saline. The PCA doses of tramadol consisted of a bolus dose of 0.1 mg/kg with a lockout interval of 20 min, no background infusion.

After the end of the surgery, patients were placed in a supine position, and anaesthesia was discontinued. Antagonism of the neuromuscular block was provided with 0.05 mg/kg neostigmine and 0.015 mg/kg atropine. The patients were extubated and then transferred to the recovery room.

The demographic data of patients (age, gender, weight, the presence of other diseases, the durations of surgery and anaesthesia, length of hospital stay) were recorded. Systolic and diastolic blood pressures (SBP, DBP), heart rate (HR), SpO_2_, visual analogue scale (VAS) scores, side effects (e.g., vomiting and nausea), complications (e.g., pneumothorax and vascular puncture), tramadol consumption and additional analgesic requirements of the patients were recorded postoperatively after 1 h in the recovery room and during the first 24 h on the urology service. Pain scores at rest were evaluated using VAS for pain (0 cm = no pain; 10 cm = worst pain imaginable). If patients had a VAS score > 4, intramuscular diclofenac sodium was administered as a rescue analgesic.

If haemodynamic changes > 20% of baseline and severe postoperative pain (VAS ≥ 7) were observed in the recovery room, the PVB was considered inadequate and excluded from analysis. Postoperative data were evaluated by an anaesthesiologist appointed as a blind independent observer.

### Statistical methods

All analyses were performed using SPSS Statistics, version 20.0, IBM. The sample size was calculated by choosing a difference of 2 points in the VAS score as the minimum expected difference between the groups. Setting α = 0.05, assuming a standard deviation of 2 points, and investigating 17 subjects for each group, one can detect a significant difference of 2 points with a power of 0.8 (two-sided hypothesis) [11]. Categorical variables are expressed as the number and percentage, whereas continuous variables are summarized as the mean and standard deviation or the median and minimum-maximum, where appropriate. The chi-square test was used to compare categorical variables between the groups. The normality of distribution for continuous variables was confirmed with the Kolmogorov-Smirnov test. Student’s t-test or the Mann-Whitney U-test was used to compare continuous variables between two groups, depending on whether the statistical hypotheses were fulfilled. To evaluate changes in the measurements obtained in the time interval, a repeated measurements analysis was applied. The values are considered statistically significant when *P* value is < 0.05.

## Results

**Fig. 1:**
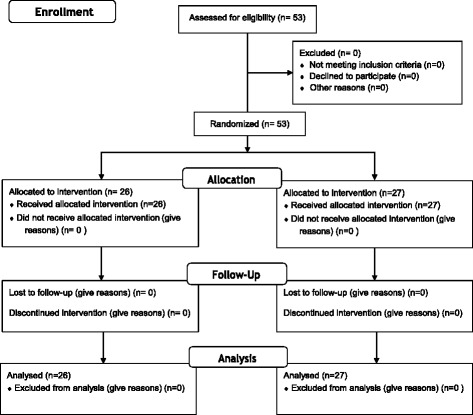
Flow diagram of the study

**Fig. 2:**
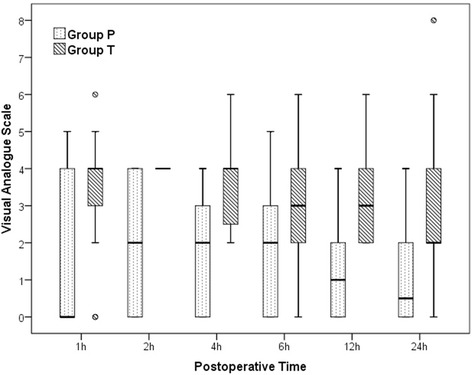
Visual analogue scale scores of the groups over postoperative time

Patient flow is summarized in the CONSORT diagram (Fig. 1). No differences were found between the two groups in terms of demographic data (Table 1). The VAS scores in group P were significantly lower than those in group T (*p* < 0.05, Fig. 2). During follow-up, two patients (7.7%) had in group P, while eight patients (29.6%) had VAS score > 4 in group T.

**Table 1:** Demographic and clinical data

	Group P (*n* = 26)	Group T (*n* = 27)	*P* values
Age (years)	41.8 ± 12.3	44.5 ± 14.0	0.45
Gender (F/M)	10/16	10/17	0.91
Weight (kg)	73.8 ± 12.7	74.5 ± 12.7	0.85
Diabetes mellitus	2(7.7%)	5(18.5%)	0.24
Hypertension	8(30.8%)	5(18.5%)	0.30
Operation time (min)	87.0 ± 36.3	94.1 ± 27.8	0.42
Duration of anaesthesia (min)	94.8 ± 36.7	100.9 ± 27.3	0.49
Hospital stay (days)	2.8 ± 1.2	3.9 ± 2.7	0.06

Data are presented as number, percentage, and mean ± standard deviation

*F* female, *M* male

In the 24-h postoperative period, total PCA tramadol consumption was significantly lower in group P than in group T (*p* < 0.001). Total PCA tramadol consumption was 77.7 ± 60.5 in group P and 142.5 ± 61.0 in group T. Two patients in group P required supplemental analgesia, while eight patients needed supplemental analgesia in group T, postoperatively (Table 2).

**Table 2:** Postoperative data

	Group P (*n* = 26)	Group T (*n* = 27)	*P* values
Nausea, n (%)	7 (26.9%)	8 (29.6%)	0.82
Vomiting, n (%)	6 (23.1%)	6 (22.2%)	0.94
Additional analgesic, n (%)	2 (7.7%)	8 (29.6%)	0.04*
Lung complications, n (%)	0	0	0.98

Data are presented as number and percentage

*p < 0.05 Group P compared to Group T

The SBP, DBP, HR and SpO_2_ values in the postoperative period were similar between the two groups. None of the patients developed complications related to the PVB. There were no significant differences between the two groups in terms of opioid-related side effects (e.g., nausea and vomiting) (Table 2).

## Discussion

We found that the patients administered multilevel paravertebral injections under ultrasound guidance had lower pain scores after PCNL. Additionally, these patients had a reduced need for tramadol IV PCA as well as rescue analgesia.

The use of the loss-of-resistance technique for PVBs in PCNL has been previously reported in the literature [5, 6, 12]. In a randomized prospective study, Ak et al. compared a PVB applied through a multilevel injection technique with morphine PCA consumption for PCNL, and they stated that thoracic PVB decreases morphine consumption and pain scores in the postoperative period [6]. These results support those of our study. However, there is a difference between the methods of the two studies. A PVB was applied by the loss-of-resistance technique in their study. We state that the VAS scores of the patients administered an ultrasound-guided PVB were significantly lower than those of patients who received tramadol PCA only during postoperative period. The total tramadol consumption was decreased by 45.5% in the group P compared with the tramadol group, and additional analgesic was administered to two patient in group P.

In adult patients, ultrasound-guided PVB has been commonly used to manage postoperative pain of breast and thoracic surgeries [13, 14]. The results of these studies showed that a PVB is an effective method to reduce postoperative pain. Similarly, a randomized controlled study of patients undergoing nephrectomy showed that the use of ultrasound-guided PVBs significantly decreased fentanyl consumption [15]. In accordance with these results, we found that ultrasound-guided PVBs provided effective analgesia in this study, thereby decreasing tramadol consumption.

In a randomized prospective study conducted by Rashwan et al., the effects of PVB, epidural and general anaesthesia were compared among patients undergoing PCNL. They evaluated both intraoperative haemodynamic changes and postoperative pain. They noted that PVB anaesthesia is more influential than epidural and general anaesthesia in terms of intraoperative haemodynamic stability and reduced analgesic consumption [12]. However, in their study, the PVBs were applied via a single injection through a catheter, using the loss-of-resistance technique before the start of surgery, which makes an adequate comparison impossible. Additionally, the accuracy of catheter placement is controversial [16]. We conclude that the ultrasound-guided multilevel paravertebral injections applied in the current study were associated with decreased VAS scores and tramadol consumption.

Paravertebral blocks have minimal effects on cardiovascular variables [8, 17]. We did not encounter a significant decrease in blood pressure or heart rate with PVBs in the postoperative period. Moreover, we found no differences in haemodynamic values between the groups. These results can be explained by the less significant sympathetic blockade and unilateral segmental nature of the block. Another positive effect reported in a systematic review was that PVBs reduce the incidence of opioid-related side effects, such as nausea and vomiting [18]. Coopey et al. claimed that preoperative PVB shortens length of hospital stay after mastectomy plus immediate reconstruction [19]. However, we did not find that PVBs decrease these side effects and length of hospital stay, probably due to the small sample size of the study.

In the current study, ultrasound-guided PVBs were performed at multiple levels, which is more extensive and reliable in terms of the radiological and sensorial distribution than single-level injection PVBs [20]. Although both single-level and multilevel PVB injections have been shown to effectively manage postoperative pain in previous studies, a recently published meta-analysis showed that compared with single-level injection, multilevel injection technique is more effective for managing acute pain during movement [13]. We did not make technical comparisons in this study. Therefore, we can only conclude that effective analgesia was provided for patients who received multilevel injections.

For practitioners, the associated complications represent the most critical drawback of utilizing a PVB, which are often attributable to multilevel injections [11]. In this respect, Naja et al. investigated the failure rate and complications following PVB among a large cohort of patients (662 patients; 620 adults, 42 children). The following rates were reported: block failure rate, 6.1%; inadvertent intravascular placement, 6.8%; epidural and/or intrathecal spread, 1.0%; pleural puncture, 0.8%; and pneumothorax, 0.5% [21]. In the present study, no complications or failed blocks were observed in any patients who received an ultrasound-guided PVB. We believe that this result is related to the application of PVBs under ultrasound guidance. To the best of our knowledge, ultrasound guidance enables direct visualization of the target nerve, needle, injected local anaesthetic and adjacent anatomical structures, thus enabling reduced incidences of complications and failed blocks [9].

However, one previous study reported complications despite the use of ultrasound. Feldzer et al. found that total spinal anaesthesia developed after applying an ultrasound-guided PVB in a patient prepared to undergo a mastectomy. In this case, an ultrasound-guided PVB was performed at the T_3_ level using the out-of-plane technique by an unsupervised trainee anaesthesiologist. The authors proposed that blocks should be done by experienced anaesthesiologists and trainees under supervision [22]. However, more important factors are which technique is used and how many levels of block are applied under ultrasound guidance. Chelly reported that a lateral-to-medial needle approach is associated with more epidural or intrathecal spread. Additionally, the application of multiple levels of local anaesthetic solution instead of a single level decreased the risk of epidural spread. Local anaesthetic volumes of 15 mL or more may increase the likelihood of epidural spread [23]. We have not experienced epidural or intrathecal spread following the ultrasound-guided PVBs that we have performed in the vertical-to-caudal direction with the in-plane technique at multiple levels.

Bupivacaine and ropivacaine at 0.5% are commonly used local anaesthetics for PVBs [24]. We injected 0.5% bupivacaine at a total dose of 15 mL (75 mg) at the T_11_-L_1_ levels. This dosage is below the recommended maximum dosage (175 mg) for major nerve blocks [25]. The duration of prolonged analgesia is subject to variability in patients with a PVB due to the use of low-dose tramadol over 24 h. It is known that the average duration of analgesia for bupivacaine is 6-12 h. The mechanism explaining the duration of prolonged analgesia remains uncertain, although analgesia is likely the effect of local anaesthetic-induced sympathectomy on pain after surgery [26].

The present study has some limitations worth noting. First, the patients in group T were not administered a sham block for ethical reasons. Therefore, the present study does not contain a control group, and only the observer was blind to the patient group allocations. Second, we did not evaluate patient and surgeon satisfaction on the efficacy of PVB on pain. However, we think that pain relief methods and their effectiveness are an important issue in terms of patient and surgeon in the postoperative period.

## Conclusion

Ultrasound-guided PVBs using bupivacaine with iv tramadol provide more effective postoperative analgesia than iv tramadol alone having no additional complications in patients undergoing PCNL. In the postoperative period, ultrasound-guided PVBs using bupivacaine was lead to lower tramadol consumption. In combination with iv tramadol in the postoperative period, ultrasound-guided PVBs with bupivacaine provides more effective analgesia than iv tramadol alone.

## Abbreviations

DBP: Diastolic blood pressure
HR: Heart rate
IV: Intravenous
PCA: Patient controlled analgesia
PCNL: Percutaneous nephrolithotomy
PVB: Paravertebral block
SBP: Systolic blood pressure
SpO_2_: Peripheral oxygen saturation
VAS: Visual analogue score
